# In vitro evaluation of the reductive carbonyl idarubicin metabolism to evaluate inhibitors of the formation of cardiotoxic idarubicinol via carbonyl and aldo–keto reductases

**DOI:** 10.1007/s00204-023-03661-7

**Published:** 2024-01-04

**Authors:** Gzona Bajraktari-Sylejmani, Julia Sophie Oster, Jürgen Burhenne, Walter Emil Haefeli, Max Sauter, Johanna Weiss

**Affiliations:** Department of Clinical Pharmacology and Pharmacoepidemiology, Heidelberg University, Medical Faculty Heidelberg, Heidelberg University Hospital, Im Neuenheimer Feld 410, 69120 Heidelberg, Germany

**Keywords:** Idarubicin, Idarubicinol, AKR, CBR, Luteolin, Ranirestat, Menadione, 2′-hydroxyflavanone

## Abstract

The most important dose-limiting factor of the anthracycline idarubicin is the high risk of cardiotoxicity, in which the secondary alcohol metabolite idarubicinol plays an important role. It is not yet clear which enzymes are most important for the formation of idarubicinol and which inhibitors might be suitable to suppress this metabolic step and thus would be promising concomitant drugs to reduce idarubicin-associated cardiotoxicity. We, therefore, established and validated a mass spectrometry method for intracellular quantification of idarubicin and idarubicinol and investigated idarubicinol formation in different cell lines and its inhibition by known inhibitors of the aldo–keto reductases AKR1A1, AKR1B1, and AKR1C3 and the carbonyl reductases CBR1/3. The enzyme expression pattern differed among the cell lines with dominant expression of CBR1/3 in HEK293 and MCF-7 and very high expression of AKR1C3 in HepG2 cells. In HEK293 and MCF-7 cells, menadione was the most potent inhibitor (IC_50_ = 1.6 and 9.8 µM), while in HepG2 cells, ranirestat was most potent (IC_50_ = 0.4 µM), suggesting that ranirestat is not a selective AKR1B1 inhibitor, but also an AKR1C3 inhibitor. Over-expression of AKR1C3 verified the importance of AKR1C3 for idarubicinol formation and showed that ranirestat is also a potent inhibitor of this enzyme. Taken together, our study underlines the importance of AKR1C3 and CBR1 for the reduction of idarubicin and identifies potent inhibitors of metabolic formation of the cardiotoxic idarubicinol, which should now be tested in vivo to evaluate whether such combinations can increase the cardiac safety of idarubicin therapies while preserving its efficacy.

## Introduction

The anthracycline drug idarubicin has been used for decades predominantly for the treatment of acute myeloid leukemia (AML) and acute nonlymphocytic leukaemia (ANLL), especially in paediatric populations. The main mechanisms underlying the antiproliferative effects of anthracyclines consist of intercalation and cross-linking of DNA and of inhibition of the topoisomerase II leading to disturbed DNA synthesis (Pommier et al. 2010; Marinello et al. 2018; Kaczorowska et al. 2020).

**Fig. 1:**
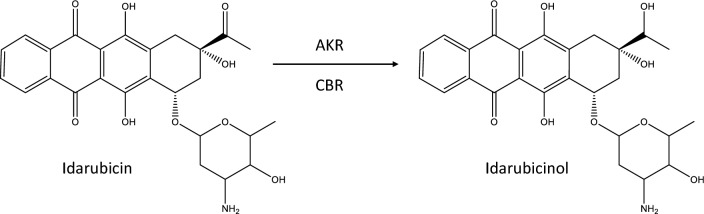
Metabolism of idarubicin to idarubicinol. Chemical structures were plotted with ChemDraw Professional Version 20.0.0.41

The main pathway for anthracycline metabolism is a two-electron reduction of the C-13 carbonyl group, producing alcohol metabolites, as depicted in Fig. 1 for idarubicin. This process is primarily catalyzed by two groups of enzymes, the superfamily of aldo–keto reductases (AKRs) and the carbonyl reductases (CBRs) belonging to the superfamily of short-chain dehydrogenases/reductases (SDRs) (Le Bot et al. 1998; Hofman et al. 2015; Koczurkiewicz-Adamczyk et al. 2022; Novotná et al. 2020).

Idarubicin is extensively metabolized to idarubicinol, which has a long half-life in the blood and peak plasma concentrations generally exceeding those of idarubicin (Crivellari et al. 2004; Tamassia et al. 1987; Gillies et al. 1987; Zanette et al. 1990; Camaggi et al. 1992; Robert et al. 1993). The main metabolising organ is the liver and idarubicin is primarily excreted as idarubicinol via the bile—only about 5% of an idarubicin dose can be found in urine, mainly as idarubicinol (Crivellari et al. 2004). Unlike other alcohol metabolites of anthracyclines, idarubicinol also has high cytotoxic activity (Ferrazzi et al. 1991; Kuffel et al. 1992; Toffoli et al. 1996 and similar potency to its parent compound (Toffoli et al. 1996). The carbonyl reduction of anthracyclines to their alcohol metabolites is catalyzed by different AKRs and CBR1 and CBR3. However, the importance of single enzymes appears to differ between the different anthracyclines and between tissues and different neoplastic entities (Bains et al. 2008, 2009, 2010, 2013; Novotna et al. 2008; Blanco et al. 2008; Kassner et al. 2008; Skarka et al. 2011; Hofman et al. 2014; Piska et al. 2021; Jo et al. 2017). In contrast to the other anthracyclines, whose metabolism has been largely elucidated, it is still unclear for idarubicin which reductase plays the main role in the conversion to idarubicinol. In addition, there is little data on whole cells or whole animals: most studies were conducted with purified or recombinant enzymes, which allows only limited conclusions to be drawn about the situation in vivo.

The main dose-limiting factor in anthracycline chemotherapy is the high risk of acute and/or late-onset cardiotoxicity, which can lead to heart failure and death (Henriksen 2018; Jong et al. 2022; Iarussi et al. 2005), limiting their therapeutic use. In childhood cancer survivors, cardiovascular complications are a leading cause for morbidity and mortality (Mulrooney et al. 2009, Armenian et al. 2015). There is a clear association between exposure to anthracyclines and cardiomyopathy risk (Blanco et al. 2012). For doxorubicin, the incidence of clinical heart failure is 5% at a cumulative dose of 400 mg/m^2^ and 48% at a cumulative dose of 700 mg/m^2^ (Swain et al. 2003). In leukemia patients treated with idarubicin, a cardiomyopathy incidence of 5% has been reported at cumulative doses between 150 and 290 mg/m^2^ (Anderlini et al. 1995). Although preclinical and clinical data indicate a lower cardiotoxicity of idarubicin compared to other anthracyclines (Goebel 1993; Robert 1993; Crivellari et al. 2004), the cardiotoxicity of idarubicin is similar when administered in amounts that exhibit equimolar myelotoxicity (Iarussi et al. 2005).

Whereas the exact mechanism of anthracycline cardiotoxicity is still under debate and seems to be multifactorial, several lines of evidence indicate that the secondary alcohol metabolites of anthracyclines play an important role, particularly in the chronic cardiotoxicity of anthracyclines (Menna et al. 2007): (1) These metabolites inhibit the ATPases that control systolic contraction and diastolic relaxation much more than their parent compounds (Boucek et al. 1987; Olson et al. 1988; Olson and Mushlin 1990; Mushlin et al. 1993). (2) Myocardial accumulation of these alcohol metabolites correlates with the development of cardiomyopathy (Stewart et al. 1993). (3) Some studies demonstrate an association of polymorphisms in the enzymes involved in the carbonyl reduction of anthracyclines and the cardiomyopathy risk: e.g., in children, homozygous carriers of the wildtype G-allele of the CBR3 polymorphism V244M (1096G > A), who reduce doxorubicin faster to doxorubicinol than the polymorphic variant, have an increased risk for cardiomyopathy at low-to-moderate doses of doxorubicin (Blanco et al 2008, 2012). (4) Studies in transgenic mice demonstrate that the overexpression of human CBR1 in the heart advances the development of doxorubicin-induced cardiotoxicity (Forrest and Gonzalez 2000), whereas mice with a null allele of CBR1 are protected from doxorubicin-induced cardiotoxicity (Olson et al. 2003). (5) Inhibition of CBR1 reduced the concentration of doxorubicinol in hearts, alleviated doxorubicin-induced cardiotoxicity in mice (Zhou et al. 2015; Jo et al. 2017), and protected cardiomyocytes against doxorubicin-induced toxicity in vitro Koczurkiewicz-Adamczyk et al. 2022).

The effect of the alcohol metabolites of anthracyclines on their cardiotoxicity suggests that inhibition of the carbonyl-reducing enzymes during anthracycline therapy may reduce the risk of cardiac side effects (Minotti et al. 1998; Plebuch et al. 2007). This approach is especially interesting for idarubicin, because inhibition of the formation of idarubicinol should have no profound impact on the efficacy of idarubicin: the metabolism to the alcohol metabolite does not represent a detoxification mechanism given the fact that idarubicinol is as effective as idarubicin itself (Toffoli et al. 1996; Yamashita et al. 2008). In the case of idarubicin, however, it is not yet clear which enzymes are most important for the formation of idarubicinol and which inhibitors might be suitable for suppressing this metabolic step and would therefore be promising candidates for clinical testing. We, therefore, investigated the formation of idarubicinol in different cell lines and its inhibition by known inhibitors of AKR1A1, AKR1B1, AKR1C3, CBR1, and CBR3 to elucidate the contribution of these reductases on idarubicinol formation.

## Materials and methods

### Materials

Cell culture media, fetal calf serum (FCS), supplements, Hank’s buffered salt solution (HBSS), HEPES, phosphate-buffered saline (PBS), menadione, ranirestat, GenElute™ Mammalian Total RNA Miniprep Kit, the Cytotoxicity Detection Kit (LDH), and the Amersham™ Hybond® P membrane were obtained from Sigma-Aldrich (Taufkirchen, Germany). Qiazol, 1 × Quantifast SYBR Green Mix and 1 × QuantiTect Hs were from Qiagen (Hilden, Germany) and the primers for the housekeeping genes were synthesized by Eurofins Genomics (Ebersberg, Germany). Human serum albumin (HSA) was purchased from Octapharma (Langenfeld, Germany). DMEM was purchased from PAN Biotech (Aidenbach, Germany). Pepstatin, aprotinin, and dimethyl sulfoxide (DMSO) were from AppliChem (Darmstadt, Germany). Pefabloc was obtained from Carl Roth (Karlsruhe, Germany). The RevertAid™ H Minus First Strand cDNA Synthesis Kit, the Absolute QPCR SYBR Green Mix, the Pierce™ BCA Protein Assay Kit, the Pierce ECL Western Blotting Substrate, the secondary HRP-conjugated anti-rabbit antibody, 2’-hydroxy-flavanone (2-OH-flavanone), and the RIPA buffer were purchased from Thermo Fisher Scientific (Waltham, MA, USA). The mouse monoclonal antibodies against β-actin (sc-47778), AKR1B1 (aldose reductase (H-6, sc-166918)), CBR1 (B-11, sc-390554), and CBR3 (E-12, sc-374393) were obtained from Santa Cruz (Heidelberg, Germany), the rabbit monoclonal antibody against AKR1C3 (ab209899) and the rabbit polyclonal antibody against AKR1A1 (ab125878) were from Abcam (Cambridge, United Kingdom). The secondary HRP-conjugated goat anti-Mouse IgG was from GE Healthcare (Chicago, IL, USA). The 4 × Laemmli protein sample buffer was purchased from Bio-Rad (Feldkirchen, Germany). The AKR1C3 human ORF-Clone and the pCMV6-Entry Mammalian Expression Vector were purchased from Origene (Rockwill, MD, USA). Fugene® HD Transfection Reagent was obtained from Promega (Madison, WI, USA). Ammonia solution (25%), and trifluoroacetic acid (TFA) were obtained from Merck (Darmstadt, Germany). Tert-butyl methyl ether (TBME) was provided by VWR International (Darmstadt, Germany). Purified water was produced using an arium® mini (Sartorius, Göttingen, Germany) ultrapure water system. The remaining reagents and solvents, methanol (MeOH), acetonitrile (ACN), and formic acid (FA) were purchased from Biosolve (Valkenswaard, The Netherlands) in the highest purity available. Idarubicin and idarubicinol were obtained from Toronto Research Chemicals Inc. (North York, Canada). Luteolin and leupeptin were purchased from Biomol (Hamburg, Germany) and daunorubicin (Daunoblastin®) from Pfizer Pharma (Berlin, Germany).

### Stock solutions

The stock solution of idarubicin was prepared by dissolving 1 mg idarubicin hydrochloride in 1 mL water for injection (1.9 mM). A stock solution of idarubicinol was prepared by dissolving 0.5 mg in 2 mL ACN/H_2_O 1/1 + 0.1% FA (0.5 mM). Daunorubicin stock solution contained 2 mg/mL daunorubicin hydrochloride in water for injection. Stock solutions of the inhibitors (100 mM) were prepared in DMSO. All stock solutions were stored in aliquots at -20 °C.

### Cell culture

MCF-7, HepG2, and HEK293 cells (all available at ATCC, Manassas, VA, USA) were cultured in DMEM with 10% FCS, 2 mM glutamine, 100 U penicillin/100 μg streptomycin, and 1% HEPES under standard cell culture conditions.

### Cytotoxicity assay

Cytotoxic effects can damage cells and thus influence the uptake of compounds. We, therefore, tested for possible cytotoxic effects of idarubicin, idarubicinol, and the inhibitors used in all cell lines using the Cytotoxicity Detection Kit according to the manufacturer’s instructions. Neither idarubicin nor idarubicinol nor the inhibitors tested revealed any short-time cytotoxic effects up to their maximum concentration used in the assays.

### Western blotting

After harvesting, cells were washed with ice-cold PBS and lysed in RIPA buffer supplemented with pepstatin (1 µg/mL), aprotinin (1 µg/mL), leupeptin (5 µg/mL), and pefabloc (1 mg/mL) as protease inhibitors. Protein quantification was conducted using the Pierce™ BCA Protein Assay Kit according to the manufacturer’s instructions. After SDS-PAGE proteins were blotted on a Hybond PVDF membrane. Western blot detections were performed using specific antibodies against AKR1A1 (1:1000), AKR1C3 (1:1000), AKR1B1 (1:200), CBR1 (1:200), and CBR3 (1:200). β-actin (1:2000) was used as a loading control. HRP-conjugated goat anti-Mouse IgG was used as secondary antibody for β-actin, AKR1B1, CBR1, and CBR3 detection. HRP-conjugated donkey anti-Rabbit IgG was used as secondary antibody for AKR1A1 and AKR1C3 detection.

Western blots were conducted at least in triplicate and bands were visualized by enhanced chemiluminescence using the Pierce ECL Western Blotting Substrate in an Azure Biosystems 600 detection system (Biozym, Hessisch-Oldendorf, Germany) and an Intas ECL ChemoStar PLUG Imager (Intas Science Imaging Instruments, Göttingen, Germany), respectively.

### Human liver samples

To check whether the HepG2 expression of the five reductases is similar to that in non-cancerous human liver samples, we compared their expression in HepG2 cells with the expression in seven human non-cancerous liver samples. The samples were obtained from the surrounding healthy tissue of resected liver metastases of rectum or colon carcinoma patients who did not receive any neoadjuvant chemotherapy before resection. The samples were obtained from the Department of Surgery of Heidelberg University Hospital and their use was approved by the Ethics Committee of the Medical Faculty of the University of Heidelberg (S-649/2012) and all patients gave their written informed consent prior to the study. Due to the very low amount of material, mRNA instead of protein was quantified.

### Quantification of mRNA expression of *AKR1A1, AKR1B1, AKR1C3, CBR1* and *CBR3* in human liver and in HepG2 cells

For RNA extraction from the cell lines, the GenElute™ Mammalian Total RNA Miniprep Kit was used. For the liver samples, a Qiazol method was applied as published previously (Shen et al. 2020). RNA was reverse transcribed to cDNA with the RevertAid™ H Minus First Strand cDNA Synthesis Kit according to the manufacturer’s instructions. mRNA expression was quantified by real-time reverse transcription (RT) polymerase chain reaction (qRT-PCR) with the LightCycler® 480 (Roche Applied Science, Mannheim, Germany) as described previously (Albermann et al. 2005). For quantifying target gene mRNA, the corresponding QuantiTect Hs primer set was used. As reference genes for normalization, *human acidic ribosomal protein (HUPO)* and *60 s ribosomal protein L13 (RPL13)* were used, for which primer sequences were published previously (Zisowsky et al. 2007).

PCR amplifications were carried out in 20 µL reaction volume containing 5 µL 1:10 diluted cDNA, 1 × Quantifast SYBR Green Mix and 1 × QuantiTect Hs or 1 × Absolute QPCR SYBR Green Mix and 0.15 µM sense and antisense primers each. Data were evaluated via calibrator-normalized relative quantification with efficiency correction using the LightCycler® 480 software version 1.5.1.62 (Roche Applied Science). Results were expressed as the target/reference ratio. Human liver samples were amplified in technical duplicates and HepG2 cells in septuplets.

### Uptake of idarubicin and metabolism to idarubicinol in different cell lines

To investigate idarubicin metabolism mediated by CBR1, CBR3, and AKRs in different cell lines, its uptake and the formation of idarubicinol was quantified in MCF-7, HepG2, and HEK293 cells.

In brief, cells were washed once with PBS after harvesting. For each sample, 1 × 10^6^ cells were suspended in 300 µL medium containing 1 µM idarubicin in 1.5 mL low-binding reaction tubes and incubated at 37 °C on a rotary shaker (450 rpm) for 10, 30, 60, 90, or 120 min. Uptake was stopped by 5 min centrifugation at 1000 × g and 4 °C. Subsequently, cells were washed twice with ice-cold HEPES buffered HBSS (HHBSS)/2% HSA before further proceeding as described in the section presenting the UPLC-MS/MS method.

### Inhibition of idarubicinol formation in different cell lines

To investigate the effects of AKR and CBR inhibitors on the formation of idarubicinol in different cell lines, intracellular concentrations of idarubicin and idarubicinol were quantified in MCF-7, HepG2, and HEK293 cells. After 5 min pre-incubation with the respective inhibitor and 30 min incubation with 1 µM idarubicin, idarubicin and idarubicinol were quantified in cell pellets as described in the section presenting the UPLC-MS/MS method. Luteolin, 2-OH-flavanone, menadione, and ranirestat were tested between 0.5 and 150 µM and ranirestat additionally at 0.05 and 0.15 µM in HepG2 cells. For calculation of the IC_50_ values, non-linear regression curves were calculated with GraphPad Prism version 9.4.1 (GraphPad Software Inc., La Jolla, CA, USA) using the four-parameter fit (sigmoidal dose–response curves with variable slope). Each experiment was conducted thrice with three technical replicates each.

### Over-expression of AKR1C3 in HEK293 and inhibition of idarubicinol formation by ranirestat

To verify inhibition of AKR1C3 by ranirestat, HEK293 cells expressing only very low amounts of AKR1C3 were transiently transfected with AKR1C3 using the AKR1C3 ORF clone. As a mock control, HEK293 cells were transfected with the empty vector pCMV6-Entry. Cells were transfected in 12-well plates after reaching 60–80% confluency using Fugene® HD at a ratio of 2:1 transfection reagent to DNA. Two days after transfection, cells were harvested and used for inhibition assays with ranirestat as described in the section before. Ranirestat was tested at a concentration of 150 µM corresponding to the concentration with the maximum inhibition of idarubicin metabolism in our previous experiments. To determine the IC_50_ value of inhibition of idarubicinol formation by ranirestat in HEK293-AKR1C3 cells, ranirestat was tested in a range from 0.015 to 150 µM. For calculation of the IC_50_ values, non-linear regression curves were calculated with GraphPad Prism version 9.4.1 (GraphPad Software Inc., La Jolla, CA, USA) using the four-parameter fit (sigmoidal dose–response curves with variable slope). The experiment was conducted thrice with two technical replicates each.

### Intracellular quantification of idarubicin and idarubicinol by ultra-high-performance liquid chromatography coupled to triple quadrupole tandem mass spectrometry (UPLC-MS/MS)

For intracellular concentration analyses, cell pellets were lysed using 100 µL of 5% aqueous NH_3_. Concentrations of idarubicin and idarubicinol in the lysates were quantified with a UPLC-MS/MS (Acquity Classic UPLC system coupled to a Xevo TQ-XS triple quadrupole mass spectrometer equipped with a Z-spray heated electrospray ionisation source; Waters, Eschborn, Germany), which was validated according to the applicable parts of the ICH M10 guideline on bioanalytical method validation and study sample analysis (ICH 2023). Calibration and quality control (QC) standards were prepared by spiking 100 µL of blank cell lysate with 25 µL of the respective standard solution. Calibration samples were prepared at 0.1, 0.3, 1, 3, 10, 30, and 100 ng/mL and QC samples were prepared independently at 0.1, 0.3, 37.5, and 75 ng/mL. Daunorubicin was used as internal standard (IS) at a sample concentration of 25 ng/mL. The assay achieved an LLOQ of 0.1 ng/mL and fulfilled the pertinent limits for accuracy and precision (± 15%, ± 20% at LLOQ) with interday and intraday accuracy of 105.6–114.0% and corresponding precision ≤ 8.1%. The IS-normalized matrix effect was within required limits (± 15%) and recovery was consistent and above 80% for all substances. Processed extracts were stable over 24 h at 10 °C covering the required time for sample measurements.

### Sample preparation

Cell pellets were lysed with 100 µL of 5% aqueous NH_3_. For the calibration standards and QCs, 25 µL of the corresponding spike solution was added. Study samples were spiked with 25 µL of blank solvent (ACN/water + 0.1% FA) for volume compensation. Subsequently, 25 µL of IS solution was added to each sample. Protein precipitation was carried out by adding 100 µL of 25% aqueous TFA. After centrifugation (16,100 × *g*, 3 min), 150 µL of the supernatant was transferred to a 96-well collection plate for injection of 20 µL onto the UPLC-MS/MS system.

### UPLC-MS/MS conditions

Chromatography was performed on an ACQUITY UPLC® Peptide BEH C18 300 Å column (1.7 µm, 2.1 × 50 mm) maintained at 40 °C using gradient elution and mobile phases consisting of water/ACN 9/1 + 0.1% FA (A) and ACN + 0.1% FA (B) with a flow rate of 0.5 mL/min. The gradient started at 5% B and was changed after 0.1 min to 40% B within 1.9 min. Then, a flushing step of 95% B for 0.5 min was performed (change to 95% within 0.1 min) before returning to initial conditions within 0.3 min and equilibration for 0.2 min. This resulted in a run time of 3 min. Initial conditions were maintained during preparation of the subsequent injection resulting in a cycle time of 4 min. The peaks of idarubicin and idarubicinol were baseline separated with retention times of 1.91 and 1.76, respectively (daunorubicin 1.78), which is essential to avoid interference of idarubicin isotopes in the idarubicinol measurements.

Mass spectrometric detection was performed in the positive ion mode with multiple reaction monitoring using argon for collision-induced dissociation. The selected mass transitions were *m/z* 498.0 → 291.0 for idarubicin at a collision energy of 12 V, *m/z* 500.0 → 291.0 for idarubicinol at a collision energy of 12 V, and *m/z* 528.0 → 321.0 for the IS daunorubicin at a collision energy of 8 V, corresponding to the identical dissociation location in the molecules. MS/MS parameters were optimized with the integrated MassLynx system software (v 4.2) and are shown in Table 1.

**Table 1:** MS/MS conditions

Parameter	Value
Capillary voltage [kV]	1.5
Source temperature [°C]	150
Desolvation temperature [°C]	600
Cone gas flow (N_2_) [L/h]	150
Desolvation gas (N_2_) [L/h]	1000
Collision gas flow (Ar) [mL/min]	0.15
Nebulizer gas flow [bar]	7
Dwell time [ms]	50

### Statistical analysis

Statistical differences in idarubicinol formation in the transfected cells were calculated using one-way ANOVA followed by Dunnett’s multiple comparison test. A *p* value < 0.05 was considered statistically significant.

## Results

### Protein expression of AKR1A1, ARK1B1, AKR1C3, CBR1, and CBR3 in MCF-7, HepG2, and HEK293 cells

**Fig. 2:**
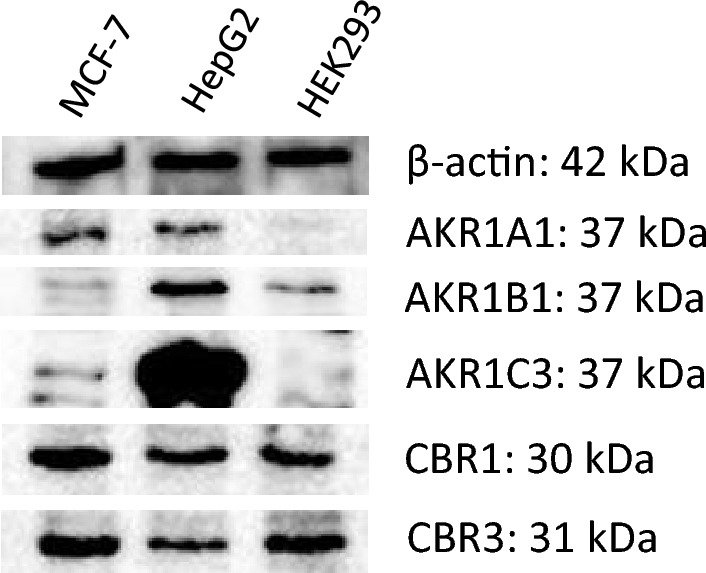
Western blot of the AKR1B1, AKR1A1, AKR1C3, CBR1, and CBR3 in HepG2, MCF-7, and HEK293 cells. β-actin served as a loading control. Depicted is one blot of a series of three for each protein investigated

Western blot data of the protein expression of the five reductases investigated revealed a different expression pattern in MCF-7, HepG2, and HEK293 cells (Fig. 2): AKR1A1 expression was generally low and nearly absent in HEK293 cells. AKR1B1 expression was highest in HepG2 cells and only weak in MCF-7 and HEK293 cells. AKR1C3 was over-expressed in HepG2 cells, but only weak in the other two cell lines. CBR1 and CBR3 expression was similar in all three cell lines with the highest expression in MCF-7 cells.

### Formation of idarubicinol in MCF-7, HepG2, and HEK293 cells

**Fig. 3:**
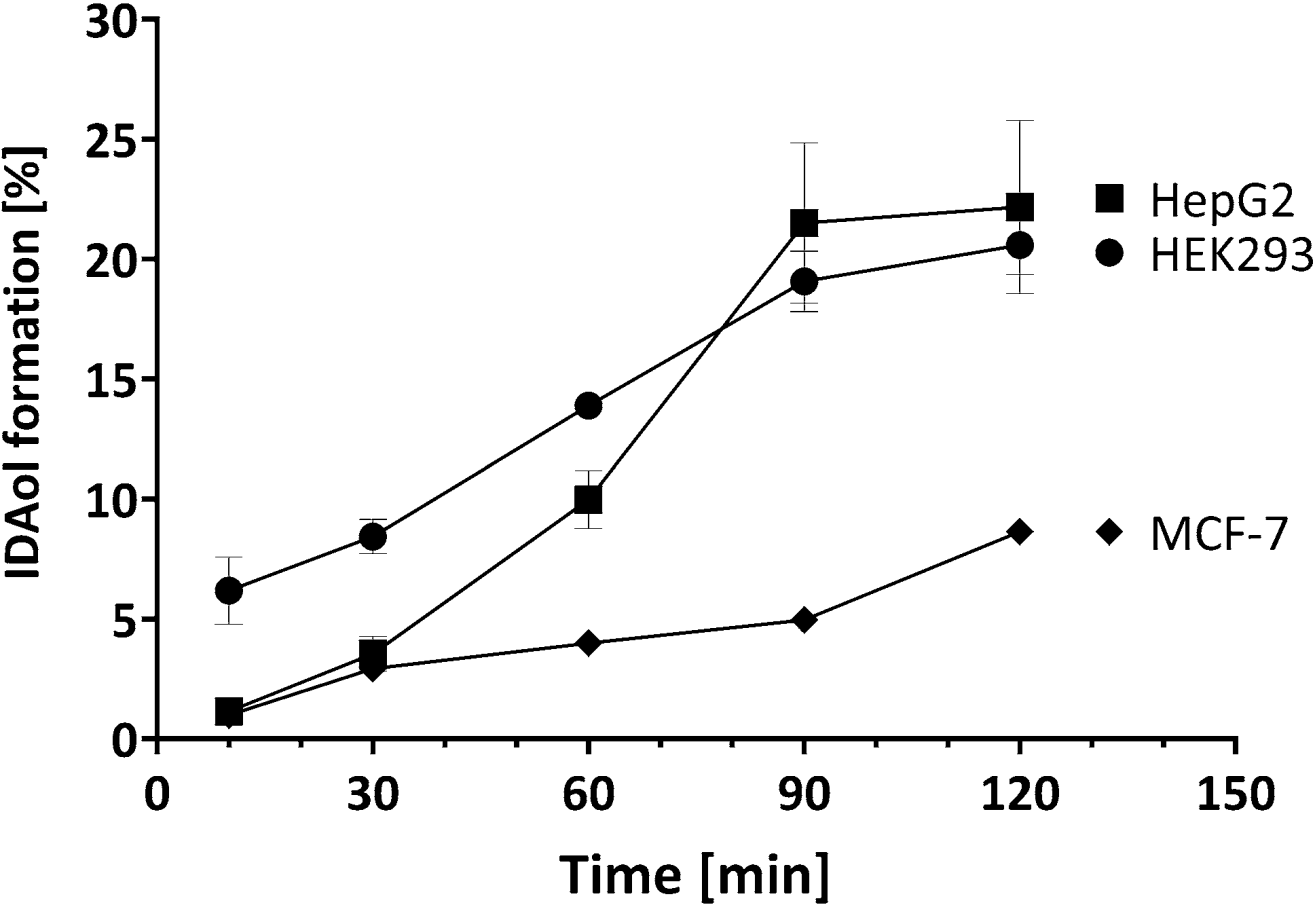
Formation of idarubicinol (%) in the different cell lines after incubation with 1 µM idarubicin. Intracellular idarubicin and idarubicinol were quantified by UPLC-MS/MS. Depicted are the results of a sextuplett. Each value represents the mean ± SEM

Idarubicinol was generated in all three cell lines, whereas the ratio between idarubicin and idarubicinol reached about 20% in HepG2 and HEK293 after 120 min of incubation and about 8.5% in MCF-7 cells (Fig. 3).

### Inhibition of idarubicinol formation by ranirestat, menadione, 2-OH-flavanone, and luteolin

To investigate which reductase might be the most important in idarubicinol formation and which inhibitor might be promising in preventing its generation, we quantified the inhibitory potency and efficacy of four known inhibitors in the three cell lines investigated.

**Fig. 4:**
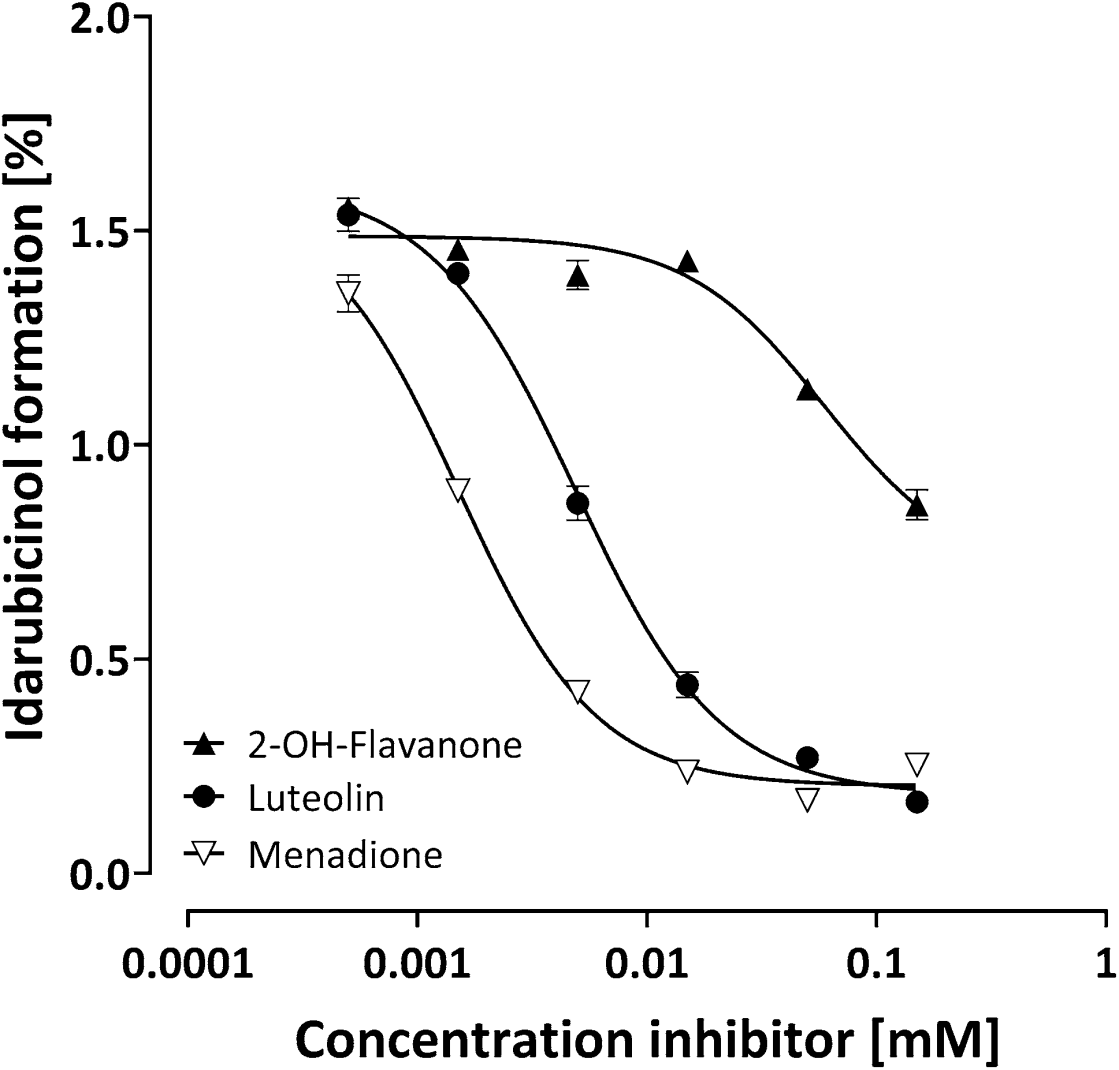
Inhibition of idarubicinol formation in HEK293 cells. Cells were pre-incubated for 5 min with the respective inhibitor before adding idarubicin (final concentration 1 µM) and incubation for further 30 min. Intracellular idarubicin and idarubicinol were quantified by UPLC-MS/MS. Depicted is the idarubicinol formation (% of idarubicin) of one experiment of a biological triplicate and each data point represents the mean ± S.E.M. of a technical triplicate

In HEK293 cells (Table 2; Fig. 4), menadione was the most potent inhibitor, followed by luteolin, whereas 2-OH-flavanone was much weaker and did not reach maximum effects up to 150 µM. Ranirestat had no effect at all, excluding a substantial contribution of AKR1B1 to the idarubicinol formation in this cell line (data not shown).

**Table 2:** Inhibition of idarubicinol formation by different inhibitors in HEK293, HepG2, and MCF-7 cells

Compound	Established inhibitor for	HEK293		HepG2		MCF-7	
		IC_50_ [µM]	% Inhibition*	IC_50_ [µM]	% Inhibition*	IC_50_ [µM]	% Inhibition*
2-OH-flavanone	AKR1C3	> 50 **	51	4.5 ± 1.2	73	48.4 ± 5.7	48
Luteolin	AKR1C3, CBR1	3.7 ± 1.0	91	34.5 ± 4.4	74	14.9 ± 2.5	86
Menadione	CBR1, CBR3	1.6 ± 0.2	86	11.9 ± 1.9	74	9.8 ± 4.5	91
Ranirestat	AKR1B1	No inhibition	No inhibition	0.4 ± 0.1	77	No inhibition	No inhibition

^*^ at the highest concentration tested. ** Inflection point of the inhibition is not reached, thus IC_50_ was not reliably calculable. AKR, aldose reductase; CBR, carbonyl reductase

**Fig. 5:**
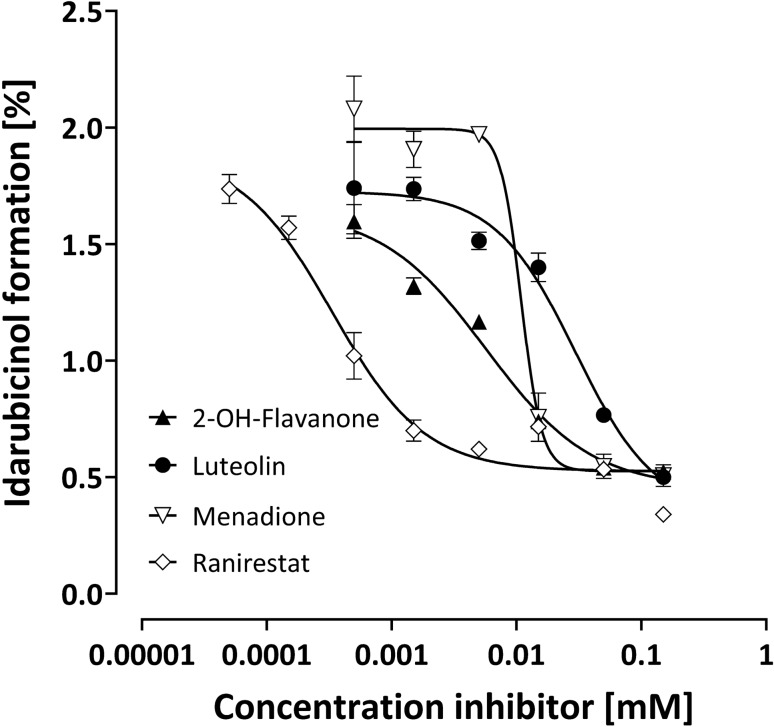
Inhibition of idarubicinol formation in HepG2 cells. Cells were pre-incubated for 5 min with the respective inhibitor before adding idarubicin (final concentration 1 µM) and incubation for further 30 min. Intracellular idarubicin and idarubicinol were quantified by UPLC-MS/MS. Depicted is the idarubicinol formation (% of idarubicin) of one experiment of a biological triplicate and each data point represents the mean ± S.E.M. of a technical triplicate

In HepG2 cells (Table 2; Fig. 5), the most potent inhibitor was ranirestat, followed by 2-OH-flavanone, menadione, and luteolin.

**Fig. 6:**
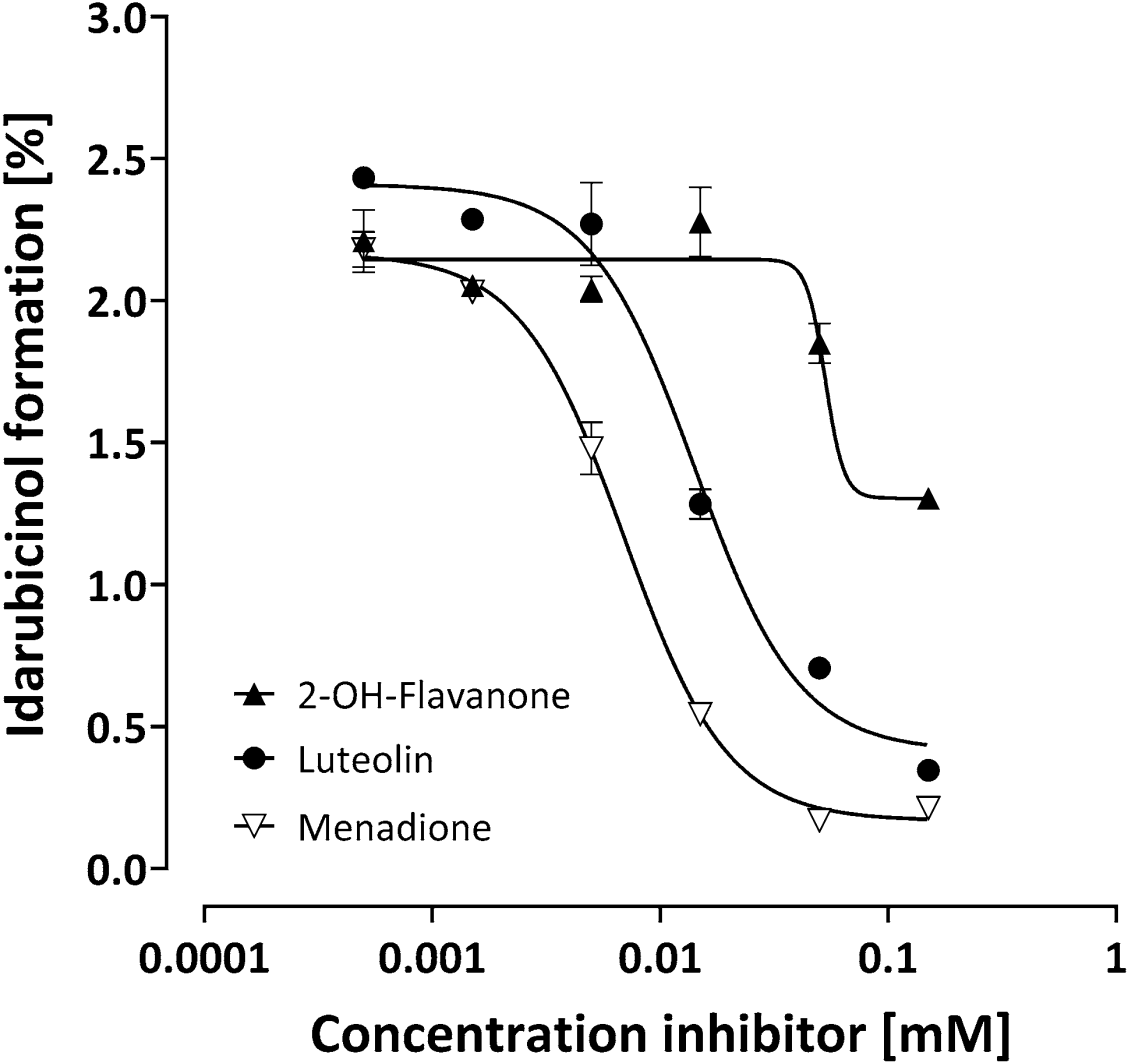
Inhibition of idarubicinol formation in MCF-7 cells. Cells were pre-incubated for 5 min with the respective inhibitor before adding idarubicin (final concentration 1 µM) and incubation for further 30 min. Intracellular idarubicin and idarubicinol were quantified by UPLC-MS/MS. Depicted is the idarubicinol formation (% of idarubicin) of one experiment of a biological triplicate and each data point represents the mean ± S.E.M. of a technical triplicate

In MCF-7 cells, menadione was most potent followed by luteolin and 2-OH-flavanone. As in HEK293 cells (Table 2; Fig. 6), ranirestat had no effects in MCF-7 cells indicating no contribution of AKR1B1 to the idarubicinol formation in this cell line (data not shown).

### Over-expression of AKR1C3 in HEK293 cells and inhibition of idarubicinol formation by ranirestat

**Fig. 7:**
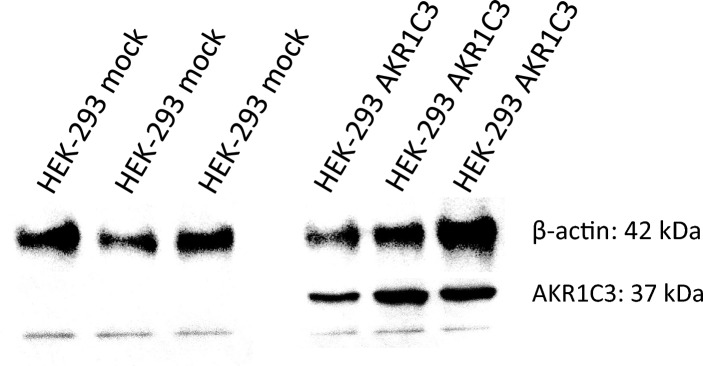
AKR1C3 western blot of HEK293 cells transfected with AKR1C3 and the empty vector (pCMV6 Entry, mock control). Depicted is the biological triplicate, β-actin was used as a loading control

**Fig. 8:**
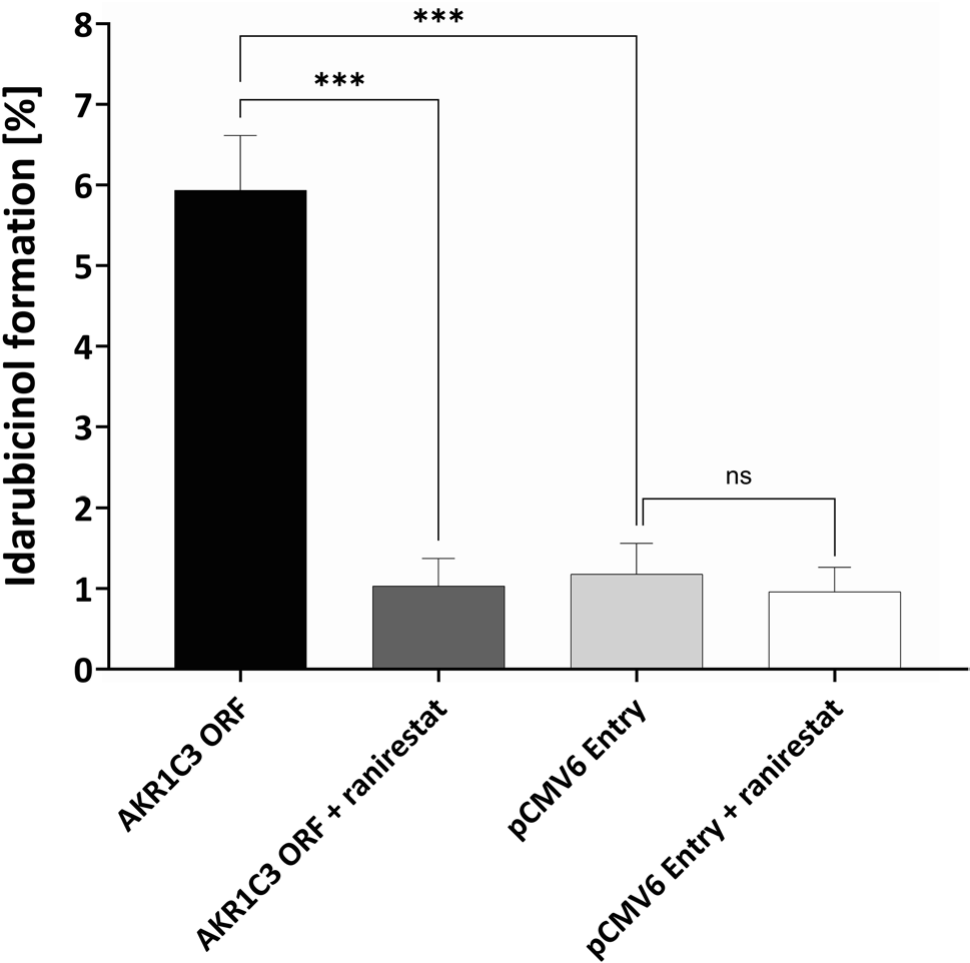
Inhibition of idarubicinol formation by ranirestat in HEK293 cells transfected with AKR1C3 or the empty vector pCMV6 Entry. Cells were pre-incubated for 5 min with 150 µM ranirestat in medium or medium alone before adding idarubicin (final concentration 1 µM) and incubation for further 30 min. Intracellular idarubicin and idarubicinol were quantified by UPLC-MS/MS. Depicted is a biological triplicate and each data point represents the mean ± S.E.M

**Fig. 9:**
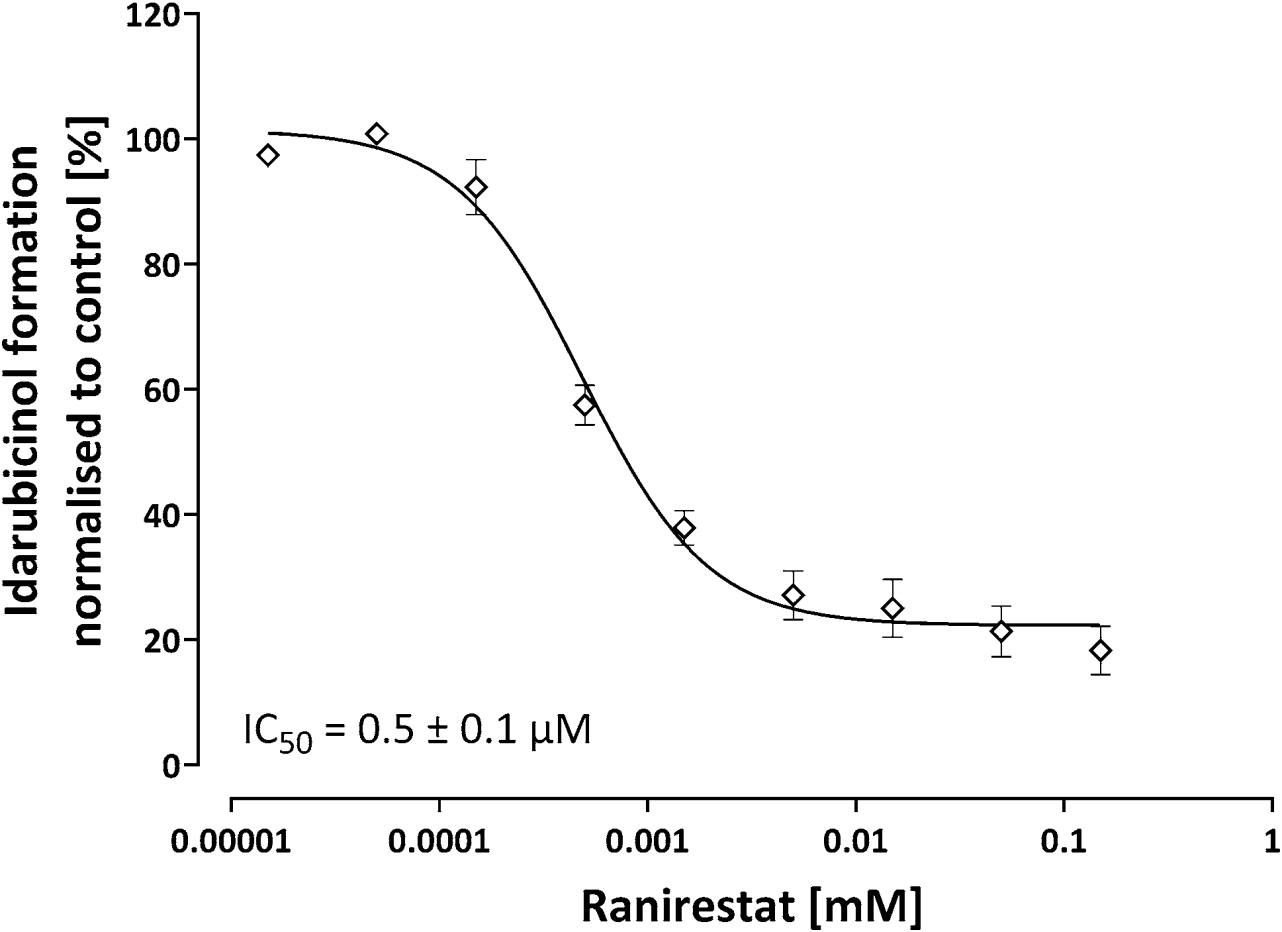
Concentration-dependent inhibition of idarubicinol formation in HEK293-AKR1C3 cells. Cells were pre-incubated for 5 min with ranirestat before adding idarubicin (final concentration 1 µM) and incubation for further 30 min. Intracellular idarubicin and idarubicinol were quantified by UPLC-MS/MS. Idarubicinol formation without addition of ranirestat was set to 100%. Depicted is the mean ± S.E.M. of a biological triplicate

HEK293 cells were successfully transfected with the AKR1C3 ORF clone (Fig. 7). As expected, the over-expression lead to a significantly higher idarubicinol formation in HEK293 cells, which could completely be reversed by 150 µM ranirestat to the level in the control cell line (Fig. 8). The IC_50_ of ranirestat for the inhibition of idarubicinol formation in HEK293-AKR1C3 cells was 0.5 ± 0.1 µM and thus similar to the IC_50_ obtained in HepG2 cells (Fig. 9; Table 2).

### Comparison of the reductase mRNA expression in human liver samples and HepG2 cells

**Fig. 10:**
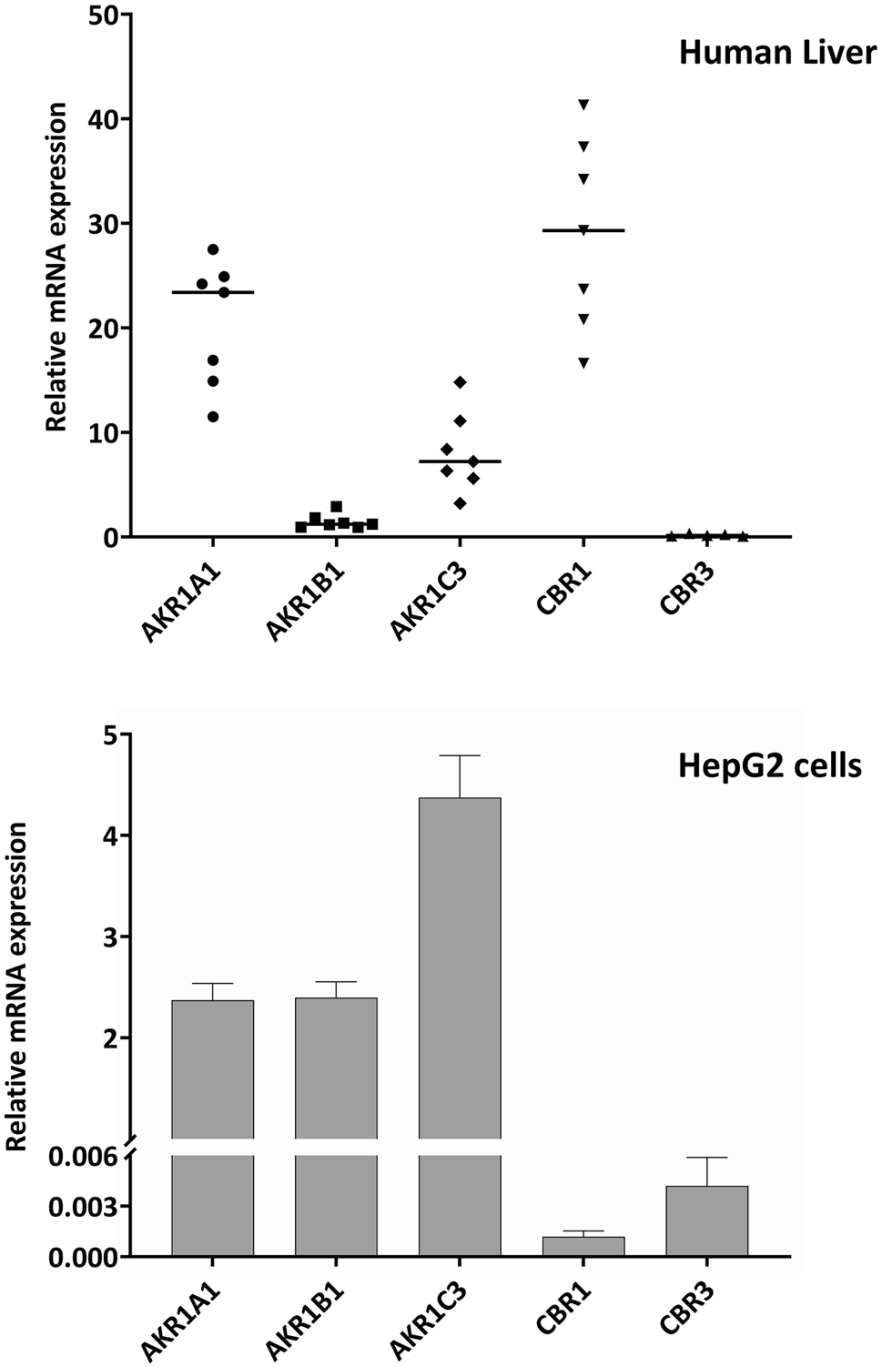
Expression of the mRNA of the five reductases in human liver samples and HepG2 cells. mRNA expression of *AKR1A1, AKR1B1, AKR1C3, CBR1*, and *CBR3* was quantified in 7 human liver samples and HepG2 cells via real-time RT-PCR and normalized to the expression of the two housekeeping genes *HUPO* and *RPL13*. Each data point for the liver samples represents the mean of a technical duplicate and the data for the HepG2 cells represent the mean ± S.E.M. for *n* = 7

To evaluate whether the expression of the AKRs and the CBR1/3 in HepG2 cells is representative for human liver, we quantified their mRNA expression in 7 human non-cancerous liver samples and compared it to the expression in HepG2 cells. As shown in Fig. 10, expression of the reductases was quite variable in human liver samples with the highest median expression of *CBR1* followed by *AKR1A1, AKR1C3*, and *AKR1B1*. Expression of *CBR3* was low and in some samples below the detection limit. In HepG2 cells, the rank order of mRNA expression was *AKR1C3* > *AKR1B1* = *AKR1A1* > *CBR3* > *CBR1*.

## Discussion

The cardiac anthracycline pool decides on the cardiac toxicity of anthracyclines and secondary alcohol metabolites like idarubicinol are supposed to play a crucial role (Boucek et al. 1987; Olson et al. 1988, 2003; Olson and Mushlin 1990; Mushlin et al. 1993; Stewart et al. 1993; Blanco et al 2008, 2012; Forrest and Gonzalez 2000; Koczurkiewicz-Adamczyk et al.; 2022; Zhou et al. 2015; Jo et al. 2017; Salvatorelli et al. 2018). Interestingly, ex vivo experiments with myocardial strips indicate that in contrast to doxorubicin and epirubicin, the cardiac anthracycline pools of daunorubicin and idarubicin mainly consist of the alcohol metabolites and for idarubicin most of the intracardial idarubicinol is taken up from plasma and not formed intracardially (Salvatorelli et al. 2018). This indicates that inhibition of systemically formed idarubicinol, which achieves high plasma concentrations (Crivellari et al. 2004) might reduce the risk of cardiotoxicity while maintaining its efficacy, because this metabolite is as effective as the parent compound in killing tumor cells (Ferrazzi et al. 1991; Kuffel et al. 1992; Toffoli et al. 1996). While biochemical experiments with isolated enzymes, in silico docking analyses, and in vitro experiments in cells over-expressing single reducing enzymes demonstrated that CBR1 and AKR1C3 are able to form idarubicinol from idarubicin (Hofman et al. 2014; Piska et al. 2021), so far, it was not clear whether other reductases are also involved, and which inhibitors might be suitable to substantially reduce idarubicinol formation. To elucidate the reductive enzymes involved, we, therefore, compared the intracellular idarubicinol formation in comparison to the intracellular idarubicin concentration (metabolic ratio) in cell lines with different expression of several AKRs, CBR1 and CBR3 and evaluated several potential inhibitors for their potency and efficacy to inhibit the formation of idarubicinol in these cell lines.

Based on protein expression data, HEK293, HepG2, and MCF-7 cells differed especially in their AKR expressions, whereas CBR1 and CBR3 were expressed in all cell lines, albeit somewhat lower in HepG2 than in the other two cell lines (Fig. 2). Most prominent was the AKR1C3 expression in HepG2 cells, which most likely explains the highest idarubicinol formation in the cell lines tested. Given the very low expression of AKR1A1, AKR1B1, and AKR1C3 in HEK-293 cells, CBR1 and CBR3 are most likely responsible for the idarubicinol formation in this cell line. Interestingly, although the enzyme configuration was similar and the CBR expression even higher, MCF-7 produced much less idarubicinol than HEK293 cells (Fig. 3). Taken together, the expression data alone do not explain the differences in idarubicinol formation among the cell lines.

To further elucidate the idarubicinol formation, we tested known inhibitors of the different AKRs and CBR1 and CBR3 that may be suitable for use as cardioprotective agents during idarubicin therapy regimens: 2-OH-flavanone, menadione, luteolin, and ranirestat.

2-OH-flavanone is a potent AKR1C3 inhibitor (Skarydova et al. 2009; Hofman et al. 2014; Verma et al. 2019) that also inhibits AKR1C1 and AKR1C2, albeit less potently (Skarydova et al. 2009). Menadione is a non-selective CBR substrate and inhibitor (Berhe et al. 2010; Maser et al. 2000). Luteolin has originally been described as a potent CBR1 inhibitor (Arai et al. 2015) but is also a weak AKR1C3 inhibitor (Skarydova et al. 2009). Ranirestat has been developed and described as a specific and potent AKR1B1 inhibitor (Kurono et al. 2001; Ishibashi et al. 2016; Bril et al. 2004). Because our experiments did suggest that ranirestat is also an AKR1C3 inhibitor, we confirmed inhibition characteristics in HEK293 cells over-expressing AKR1C3.

In our experiments, 2-OH-flavanone inhibited idarubicinol formation with different potency and efficacy that correlated well with the extent of AKR1C3 expression. In contrast, ranirestat had no effect in HEK293 and MCF-7 cells with only very low expression of AKR1B1 (Table 2), excluding that this enzyme plays a significant role in idarubicinol formation. Surprisingly, in HepG2 cells with very high AKR1C3 expression, ranirestat potently inhibited idarubicinol formation (Table 2; Fig. 5) indicating that ranirestat is also an AKR1C3 inhibitor, which we were able to confirm.

Luteolin potently and effectively inhibited idarubicinol formation in HEK293 and MCF-7 cells (Figs. 4, 6), as did menadione, suggesting that in these cells CBR1/3 play the main role in idarubicinol formation. Nevertheless, menadione also inhibited idarubicinol formation in HepG2 cells with high efficacy, indicating that menadione is able to inhibit AKR1C3 as well. Additionally, due to the fact that ranirestat only efficiently inhibited idarubicinol formation in HepG2 cells with high AKR1C3 expression, luteolin most likely is also a weak inhibitor of CBR1/3.

One still open question is which AKRs and/or CBR are most important for the systemic formation of idarubicinol. While the obtained results make precise conclusion on the contribution to idarubicin metabolism challenging, our data verify that AKR1C3 metabolizes idarubicin and indicates that CBR1/3 also contribute to the formation of idarubicinol. Further, it may be assumed that AKR1C3 is somewhat more efficacious in metabolizing idarubicin compared to CBR1/3, because of the similar inhibition efficacy of 2-OH-flavanone, menadione, and ranirestat, which is only possible if the contribution of CBR1/3 compared to AKR1C3 is low. However, the expression levels of CBR1/3 are apparently lower in this cell line. In addition, we cannot exclude that further reductases not addressed in this study contribute to the formation of idarubicinol possibly explaining, why this metabolic step was not completely inhibited by any of the inhibitors applied.

As the liver is the main metabolising organ for idarubicin (Crivellari et al. 2004), it might be worth having a closer look at the data obtained in the hepatic HepG2 cells. In these cells, the highest idarubicinol formation was observed, which could be suppressed to around 25% of initial values with AKR1C3 and CBR inhibitors. However, the expression of the different AKRs and CBR1/3 in this tumor cell line is obviously not representative for the human liver: healthy human liver expressed much more CBR1 and less AKR1C3 mRNA than the neoplastic HepG2 cells (Fig. 10), matching previous data postulating that CBR1 is the predominant doxorubicin reductase in human liver (Kassner et al. 2008) and demonstrating that AKR1C3 is over-expressed in hepatocellular carcinoma (Zhu et al. 2021; Zhou et al. 2021; Zheng et al. 2022; Pan et al. 2022). Nevertheless, having in mind that the more relevant protein expression data do not completely match the mRNA expression data (as can be seen for HepG2 cells: cf. Figures 2 and 10), it is reasonable to assume that inhibition of AKR1C3 and/or CBR1 in humans under chemotherapy with idarubicin would substantially reduce the systemic formation of idarubicinol, which appears to be the main source of the intracardial pool (Salvatorelli et al. 2018). Therefore, AKR1C3 and CBR inhibitors might be useful adjuvants in idarubicin therapy possibly reducing the risk of cardiotoxicity. In our experiments, luteolin, ranirestat and 2-OH-flavanone did substantially reduce formation of idarubicinol, and therefore, might be feasible candidates for reducing anthracycline cardiotoxicity. Further, these substances have additional characteristics that render them suitable candidates for this purpose. Beyond its AKR1C3- and CBR-inhibiting properties, luteolin acts as an anti-oxidant, scavenger, anti-inflammatory, and UV-protecting drug and has, therefore, multiple health-promoting effects and is e.g. applied against tumors, diverse inflammations, Alzheimer’s disease, Parkinson’s disease, and Long-COVID (Tuorkey 2016; Luo et al. 2017; Aziz et al. 2018; Imran et al. 2019; Theoharides et al. 2021; Siddique 2021; Daily et al. 2021). Menadione is already tested as an adjuvant in several tumor entities and chemotherapy regimens Gul et al. 2022). 2-OH-flavanone is a natural flavonoid present in several vegetables and fruits (Bailly 2021) and already tested against several kinds of cancers (Cherian et al. 2022). Although the potency for inhibition of AKR1B1 is higher (Kurono et al. 2001; Matsumoto et al. 2009), therapeutic plasma concentrations of ranirestat, which is currently under development for the treatment of diabetic neuropathy (Bril et al. 2006; Sekiguchi et al. 2019; Itou et al. 2020) are in the micromolar range (Itou et al. 2020) and might, therefore, be high enough to efficaciously inhibit AKR1C3.

In contrast to other anthracyclines, where inhibition of the formation of alcohol metabolites via AKR or CBR increases efficacy (Liu et al. 2023; Verma et al. 2016, 2019; Jo et al. 2017; Piska et al. 2021; Koczurkiewicz-Adamczyk et al. 2022), this is most probably not the case for idarubicin, because idarubicinol is as cytotoxic as the parent compound. Interestingly, inhibition of AKR1C3 has overcome tumor resistance also towards idarubicin (Hofman et al. 2014), which is possibly caused by modulation of resistance mechanism mediated by AKR1C3, which are not associated with the metabolism of anthracyclines. Some of them are: (1) AKR1C3 promotes hormonal cancers progression by increasing local androgen and estradiol formation (Auchus 2004; Sharifi & Auchus 2012); (2) AKR1C3 activates the anti-apoptosis phosphatase and tensin homolog (PTEN)/Akt pathway (Zhong et al. 2015);; (3) AKR1C3 promotes the phosphorylation of AKT (Zheng et al. 2022); (4) AKR1C3 mediates the activation of nuclear factor kappa-light-chain-enhancer of activated B cells (NF-κB) and signal transducer and activator of transcription 3 (STAT3) (Zhou et al. 2021).

There are several limitations of our study worth to be mentioned: (1) we only investigated inhibition of the idarubicinol-formation in a hepatic tumor cell line and not in primary cells. To assess, whether systemic formation of idarubicinol can be substantially inhibited e.g. by ranirestat, these experiments should be repeated in primary cells or liver organoids or finally in animal experiments before conducting a clinical study. (2) We only investigated the expression of selected AKRs and CBR1/3 and the inhibition of idarubicinol formation by inhibitors of them. We cannot exclude that other reductases as well might contribute to idarubicinol formation in HEK293, HepG2, and MCF-7 cells including the new microsomal carbonyl reductase, which has been demonstrated to reduce idarubicin to idarubicinol (Skarka et al. 2011). (3) We did not study idarubicinol formation in cardiomyocytes, which also express reductases (Koczurkiewicz-Adamczyk et al. 2022; Salvatorelli et al. 2018; Keith et al. 2009; Liang et al. 2021). (4) We did not examine whether inhibition of idarubicinol-formation indeed reduces cardiotoxic side effects of idarubicin. However, this can only be reliably assessed in an animal or clinical study.

## Conclusions

In conclusion, our study underlines the importance of AKR1C3 and CBR1 for the reduction metabolism of idarubicin and identifies inhibitors which substantially inhibit the formation of the cardiotoxic idarubicinol and might be used in vivo in combination with idarubicin to increase the safety of the therapy while likely preserving its efficacy.

## Data Availability

The data will be made available upon request.
